# Ghrelin Axis Reveals the Interacting Influence of Central Obesity and Hypertension

**DOI:** 10.3389/fendo.2018.00534

**Published:** 2018-09-12

**Authors:** Angus P. Yu, Felix N. Ugwu, Bjorn T. Tam, Paul H. Lee, Christopher W. Lai, Cesar S. C. Wong, Parco M. Siu

**Affiliations:** ^1^School of Public Health, Li Ka Shing Faculty of Medicine, The University of Hong Kong, Pokfulam, Hong Kong; ^2^Department of Health Technology and Informatics, Faculty of Health and Social Sciences, The Hong Kong Polytechnic University, Kowloon, Hong Kong; ^3^Department of Health, Kinesiology and Applied Physiology, Concordia University, Montreal, QC, Canada; ^4^School of Nursing, Faculty of Health and Social Sciences, The Hong Kong Polytechnic University, Kowloon, Hong Kong

**Keywords:** ghrelin, obestatin, growth hormone, central obesity, hypertension

## Abstract

**Objective:** This study aimed to investigate how central obesity and hypertension modulate unacylated ghrelin (UnAG), acylated ghrelin (AG), obestatin, growth hormone (GH), and the ratios of UnAG/obestatin, AG/obestatin, and total ghrelin/obestatin.

**Methods:** Circulatory abundances of UnAG, AG, obestatin and GH were determined in 387 Hong Kong Chinese female adults with age between 24 to 86 years based on a 2 × 2 factorial design of hypertension (blood pressure ≥140/90 mmHg) and central obesity (waist circumference or WC ≥80 cm). Participants were categorized as neither hypertensive nor centrally obese (NHNO; *n* = 105), hypertensive but not centrally obese (HNO; *n* = 102), centrally obese but not hypertensive (NHO; *n* = 74) and hypertensive and centrally obese (NO; *n* = 106). Pearson's correlation analyses were performed to detect the association between the peptides examined with WC and blood pressure. The main and interaction effects of hypertension and central obesity were examined by generalized estimating equations analyses.

**Results:** Correlation analyses revealed that systolic blood pressure was negatively correlated with AG/obestatin, UnAG/obestatin and total ghrelin/obestatin ratios, AG, total ghrelin, and GH, while diastolic blood pressure was negatively correlated with UnAG/obestatin, total ghrelin/obestatin ratios, and GH. WC was negatively correlated with AG/obestatin, UnAG/obestatin, and total ghrelin/obestatin ratios, UnAG, AG, total ghrelin, GH, and obestatin. Interaction effects of hypertension and central obesity were observed on UnAG/obestatin, AG/obestatin and total ghrelin/obestatin ratios, and obestatin. Obestatin in NHO group was significantly higher compared to NHNO and HO groups. UnAG/obestatin, AG/obestatin, and total ghrelin/obestatin ratios were higher in NHNO group compared to HNO and HO groups. Main effects of central obesity and hypertension were observed in UnAG, total ghrelin and GH. The HO group manifested the lowest level of UnAG, total ghrelin and GH among all the groups studied. Main effect of hypertension was observed on AG, suggesting that hypertensive individuals exhibited lower levels of AG regardless of central obesity.

**Conclusion:** Circulatory ghrelin gene products and GH exhibit different modes of modulation in response to the co-manifestation of multiple cardiovascular risk factors compared with a single risk factor alone.

## Introduction

Obesity and hypertension are unfavorable conditions that increase morbidity and mortality. Central obesity (waist circumference ≥90 cm for male; ≥80 cm for female) and hypertension (blood pressure ≥140/90 mmHg) are two of the cardiometabolic risk factors of metabolic syndrome that predispose individuals to cardiovascular diseases and type 2 diabetes mellitus. The 26-year follow-up research of the Framingham Heart Study, which proposed initially the association between hypertension and obesity has led to intense research in obesity-related hypertension (1). Obese children have been demonstrated to be 3-fold more susceptible to the development of hypertension compared to their lean counterparts. Likewise, high blood pressure in children was associated with increased prevalence of adiposity (2). The notion that obesity promotes the development of hypertension was further supported by a joint research conducted by the European Society of Hypertension and the European Society of Cardiology, which revealed a strong correlation between hypertension and excess body weight in adults (3). One of the linking hypotheses is that obesity-related endothelial dysfunction and the functional abnormalities of the kidney are common pathologies of hypertension.

Targeting the ghrelin signaling pathway is thought to decipher the mechanisms underlying the association between obesity and hypertension (4) and carry therapeutic significance in the management of energy homeostasis and cardiovascular function (5). The ghrelin gene encodes three peptides including unacylated ghrelin (UnAG) as the predominant form of circulating ghrelin, acylated ghrelin (AG) and obestatin. Previous studies suggested that UnAG exhibits antagonistic effect to AG while having its own independent function, such as regulating glucose metabolism (6). Stimulation of food intake by AG (7) is known to be antagonized by the administration of UnAG (8) by suppressing the AG-induced neuronal activity in the brain (9). Interestingly, it has been shown in animal model that chronic intravenous infusion of acylated ghrelin promoted the increase in abdominal white adipose tissue volume, suggesting the involvement of AG in fat distribution. Despite that obestatin was initially identified as an anorexigenic peptide (10), its role in suppressing food intake remains controversial (11). However, it is suggested that the balance of ghrelin and obestatin is associated with pathological conditions including anorexia nervosa and obesity (12).

In addition to the roles of ghrelin gene products in the regulation of food intake, ghrelin and obestatin are implicated in several disorders namely obesity, type 2 diabetes and cardiovascular diseases (12–14). Previous studies have shown that the circulating levels of ghrelin gene products and the ghrelin/obestatin ratios were disrupted in obese individuals. The circulating level of total ghrelin in individuals with obesity has been demonstrated to be lowered when compared with healthy subjects with normal body weight (15). Although increased plasma obestatin was observed in concomitant with reduced plasma ghrelin and ghrelin/obestatin in obese women (16), a meta-analysis has indicated that obestatin, total ghrelin, and AG were significantly decreased in obese population (17). The concept that the disrupted balance of ghrelin gene products in the blood is associated with the development of hypertension is supported by studies exhibiting that the fasting total ghrelin, and total ghrelin/obestatin ratio were lowered in hypertensive adults (18). Intriguingly, the circulating levels of total ghrelin and obestatin were found to be lower in obese adults with hypertension compared with their normotensive counterparts (19). Given that AG is known to stimulate the release of GH from the pituitary gland, accumulating evidence has unanimously indicated a potential interplay of the ghrelin-GH pathway in the development of obesity and hypertension. In congruent with earlier studies showing that GH intervention reduced diastolic blood pressure and waist circumference in centrally obese men (20), GH supplementation has been demonstrated to reduce systolic blood pressure and improve cardiovascular function in rats exposed to adverse prenatal or postnatal conditions (21). It has been recently reported that the infusion of AG, but not UnAG, decreased blood pressure in healthy humans (22). Furthermore, GH intervention in children diagnosed with Prader-Willi syndrome retarded the progression of obesity, reduced blood pressure, and improved the metabolic profile but failed to abolish hyperphagia (23); which is in support of the notion that the association between low GH level and obesity can be ascribed to the reduction of AG in obese individuals (24). Nevertheless, the researches investigating the relationship between obestatin and the development of obesity and hypertension have yielded mixed results (13). Collectively, decreased circulating levels of ghrelin and GH are apparently associated with hypertension and obesity, while the influence of hypertension and obesity on obestatin level remains controversial.

There are ample evidences that ghrelin gene products and GH can be affected by hypertension or central obesity alone. However, studies addressing the interaction between hypertension and obesity on ghrelin gene products and GH are lacking. Taken into consideration that the antagonistic properties among the ghrelin gene products, it is essential to characterize the specific form of ghrelin, and the ratios of each ghrelin form/obestatin associated with the regulation of body visceral fat mass and blood pressure. The present study was also the first attempt to examine the interacting influence of hypertension and central obesity on circulatory UnAG, AG, obestatin, total ghrelin, ratios of ghrelin/obestatin, and GH.

## Methods

### Subjects

This study was a follow-up of our previous work with participants screened for metabolic syndrome between November 2010 and August 2013 (25). Female subjects were included in the present study because of the established gender differences in AG, total ghrelin, obestatin and GH (26–29). Besides, by selecting only female subjects, the current study attempted to interpret the interaction between hypertension and central obesity without the complications due to gender effect as previously demonstrated (30). In this study, fasting sera of 387 Hong Kong Chinese female adults within the age range of 24–86 years were retrieved from a total of 1,492 archived samples. Participants were categorized into 4 groups: NHNO—no hypertension no central obesity (*n* = 105), HNO—with hypertension but no central obesity (*n* = 102), NHO—without hypertension but with central obesity (*n* = 74) or HO—with hypertension and central obesity (*n* = 106) in accordance to the American Heart Association and National Cholesterol Education Program (NCEP)-ATP III. Individuals whose systolic and diastolic blood pressure equal or exceed 140 and 90 mmHg, respectively, were considered hypertensive. Central obesity was defined as waist circumference exceeding 80 cm (31, 32). All subjects had normal fasting triglycerides, blood glucose, and HDL-C based on NCEP-ATP III diagnostic criteria. Medical history was inquired to assure the absence of any pre-existing eating disorders. A 2 × 2 factorial research design was adopted to evaluate the interaction between hypertension and central obesity. Blood pressure was determined by an electronic blood pressure monitor (Accutorr Plus, Datascope). Waist circumference was measured with an inelastic measuring tape. All participants were fasted for at least 10 h prior to phlebotomy. Sera samples were then separated, aliquoted and stored at −80°C until measurements of UnAG, AG, obestatin, and GH. Human research ethics approval was obtained from the human subject ethics subcommittee of the Hong Kong Polytechnic University (ethics approval number: HSEARS20150203002) and written informed consent was obtained from all participating subjects.

### Peptide measurements

UnAG and AG ELISA kits were purchased from BioVendor®–Laboratiorni medicina a.s., Karasek, Czech Republic (RA194063400R and RA194062400R, respectively). Human obestatin kits were purchased from RayBiotch®, Norcross, USA (EIA-OBS). Human growth hormone kits were purchased from BioVendor—Laboratiorni medicina a.s., Karasek, Czech Republic (RCD017R). All protocols were in accordance with manufacturers' recommendations.

### Statistical analysis

Values are expressed as mean ± standard deviation. This study employed a 2 × 2 (hypertension × obesity) factorial design. Generalized estimating equations (GEE) is a commonly used method with less stringent requirement on normality assumption for factorial analysis. The main effects of hypertension and central obesity and the interaction effect of hypertension and central obesity on UnAG, AG, total ghrelin, obestatin, ratios of UnAG/obestatin, AG/obestatin, and total ghrelin/obestatin and GH were analyzed by GEE. Pearson's correlation was employed to detect the correlation between parameters. Statistical differences among the four individual groups were determined by Kruskal-Wallis H Test followed by Dunn-Bonferroni *post hoc* tests. Statistical significance was accepted at *p* < 0.05. All statistical procedures were conducted using the Statistical Package for the Social Sciences (SPSS) version 24 for Windows.

## Results

### Demographic characteristics of participants

The demographic characteristics of participants were listed in Table 1. Chi-square test revealed that the distribution of age category among the four groups were different (*p* = 0.001) (Table 1). The age of participants with hypertension were older than those with normal blood pressure. It was observed that the age of HNO and HO groups were older than that of NHNO and NHO groups (HNO vs. NHNO, *p* = 0.001; HNO vs. NHO, *p* = 0.001; HO vs. NHNO, *p* = 0.001; HO vs. NHO, *p* = 0.001) (Table 1). The HNO and HO groups had significantly higher systolic blood pressure and diastolic blood pressure compared with the other two groups (for both systolic blood pressure and diastolic blood pressure: HNO vs. NHNO, *p* = 0.001; HNO vs. NHO, *p* = 0.001; HO vs. NHNO, *p* = 0.001; HO vs. NHO, *p* = 0.001) while NHO and HO had a significantly higher waist circumference compared with the other two groups (NHO vs. NHNO, *p* = 0.001; NHO vs. HNO, *p* = 0.001; HO vs. NHNO, *p* = 0.001; HO vs. HNO, *p* = 0.001).

**Table 1:** Demographic characteristics of participants.

	**NHNO**	**HNO**	**NHO**	**HO**
Hypertension (SBP ≥ 140 mmHg or DBP ≥ 90 mmHg)	–	+	–	+
Central obesity (waist circumference ≥80 cm)	–	–	+	+
Number of subjects	105	102	74	106
Age	50.4 ± 6.1	64.1 ± 10.1^*^^#^	49.8 ± 6.4	62.9 ± 10.4^*^^#^
**NUMBER OF PARTICIPANTS IN DIFFERENT AGE GROUP**				
Young adult (age 18–35)	2 (1.9%)	0 (0%)	1 (1.3%)	0 (0%)
Middle-age adult (age 36–55)	85 (80.9%)	20 (19.6%)	59 (79.7%)	26 (24.5%)
Older adult (age >55)	18 (17.1%)	82 (80.4%)	14 (18.9%)	80 (75.5%)
**METABOLIC PARAMETERS**				
SBP (mmHg)	112.39 ± 9.79	161.26 ± 14.28^*^^#^	115.89 ± 8.12	157.90 ± 14.96^*^^#^
DBP (mmHg)	68.96 ± 6.43	79.66 ± 11.21^*^^#^	71.89 ± 7.20	81.88 ± 11.57^*^^#^
WC (cm)	72.57 ± 4.29	73.33 ± 4.60	86.26 ± 5.03^*^^†^	87.37 ± 5.69^*^^†^
GLU (mmol/L)	4.83 ± 0.33	4.91 ± 0.33	4.83 ± 0.34	4.98 ± 0.33
HDL (mmol/L)	1.76 ± 0.30	1.69 ± 0.32	1.71 ± 0.28	1.62 ± 0.26
TG (mmol/L)	0.96 ± 0.26	1.01 ± 0.27	1.05 ± 0.31	1.12 ± 0.28

*SBP, Systolic blood pressure; DBP, Diastolic blood pressure; WC, Waist circumference; GLU, Fasting blood glucose; HDL, High density lipoprotein-cholesterol; TG, Triglycerides*;

* *Significantly different from NHNO, p < 0.001*,

# *Significantly different from NHO, p < 0.001*,

† *Significantly different from HNO, p < 0.001*.

### Correlation analysis

**Figure 1:**
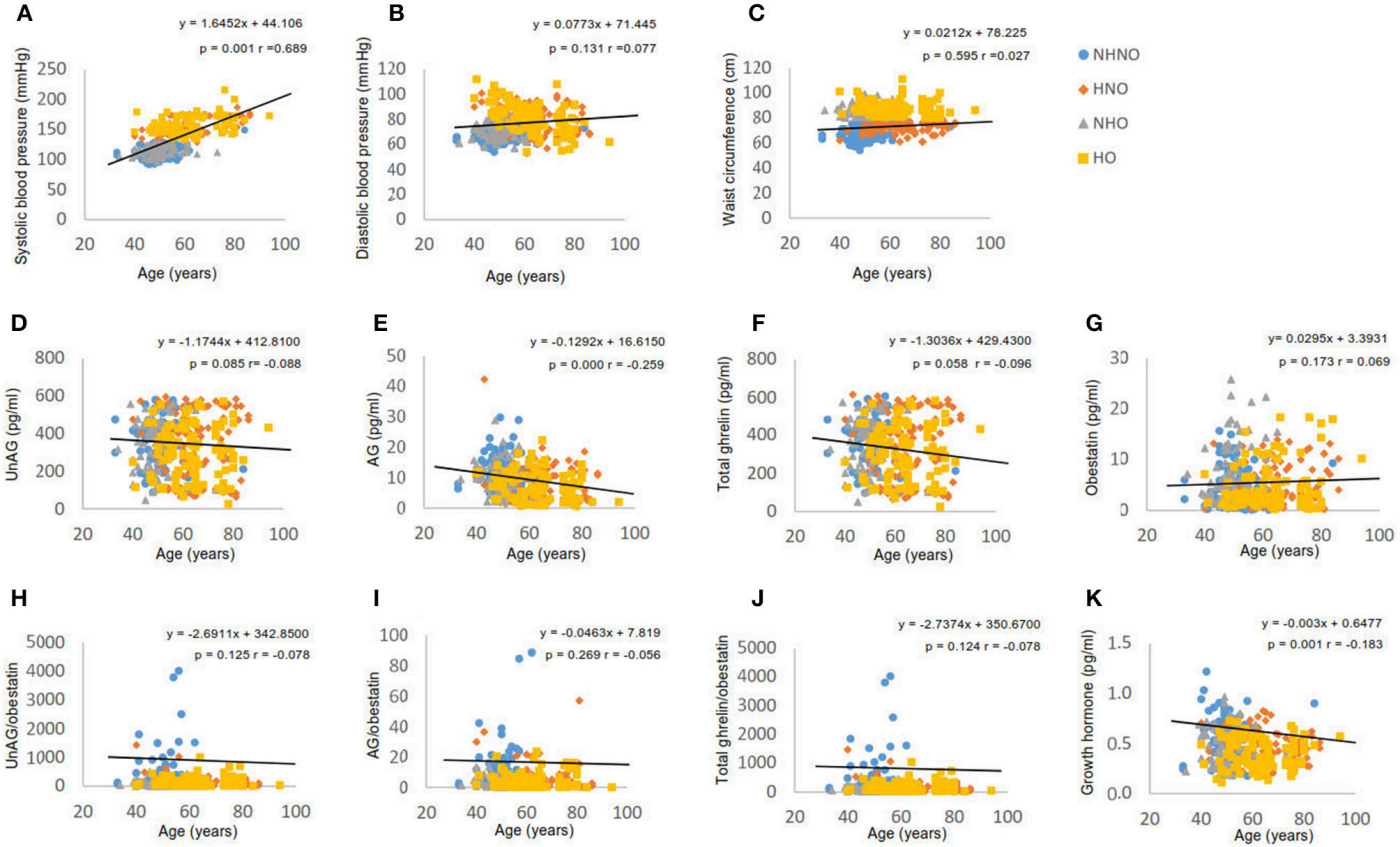
Correlation analyses of age to waist circumference, blood pressure, ghrelin gene products, growth hormone and ratios of two forms of ghrelin to obestatin. **(A)** Correlation between age and systolic blood pressure. **(B)** Correlation between age and diastolic blood pressure. **(C)** Correlation between age and waist circumference. **(D)** Correlation between age and serum level of unacylated ghrelin. **(E)** Correlation between age and serum level of acylated ghrelin. **(F)** Correlation between age and serum level of total ghrelin. **(G)** Correlation between age and serum level of obestatin. **(H)** Correlation between age and the ratio of unacylated ghrelin to obestatin in serum. **(I)** Correlation between age and the ratio of acylated ghrelin to obestatin in serum. **(J)** Correlation between age and the ratio of total ghrelin to obestatin in serum. **(K)** Correlation between age and serum level of growth hormone. Pearson's correlation analyses were performed. Statistical significance was accepted at *p* < 0.05. Circle refers to the data of non-hypertensive non-centrally obese subjects. Rhombus refers to the data of hypertensive non-centrally obese subjects. Triangle refers to the data of non-hypertensive centrally obese subjects. Square refers to the data of hypertensive centrally obese subjects.

The age of participants showed a moderate positive correlation with systolic blood pressure (*p* = 0.001, *r* = 0.698) (Figure 1A), but not with diastolic blood pressure (Figure 1B) or waist circumference (Figure 1C). Significant negative correlations were observed between age and AG (*p* = 0.001, *r* = −0.259) (Figure 1E), and between age and GH (*p* = 0.001, *r* = −0.183) (Figure 1K). However, UnAG (Figure 1D), total ghrelin (Figure 1F), obestatin (Figure 1G), UnAG/obestatin ratio (Figure 1H), AG/obestatin ratio (Figure 1I) and total ghrelin/obestatin ratio (Figure 1J) did not exhibit any correlation with age.

**Figure 2:**
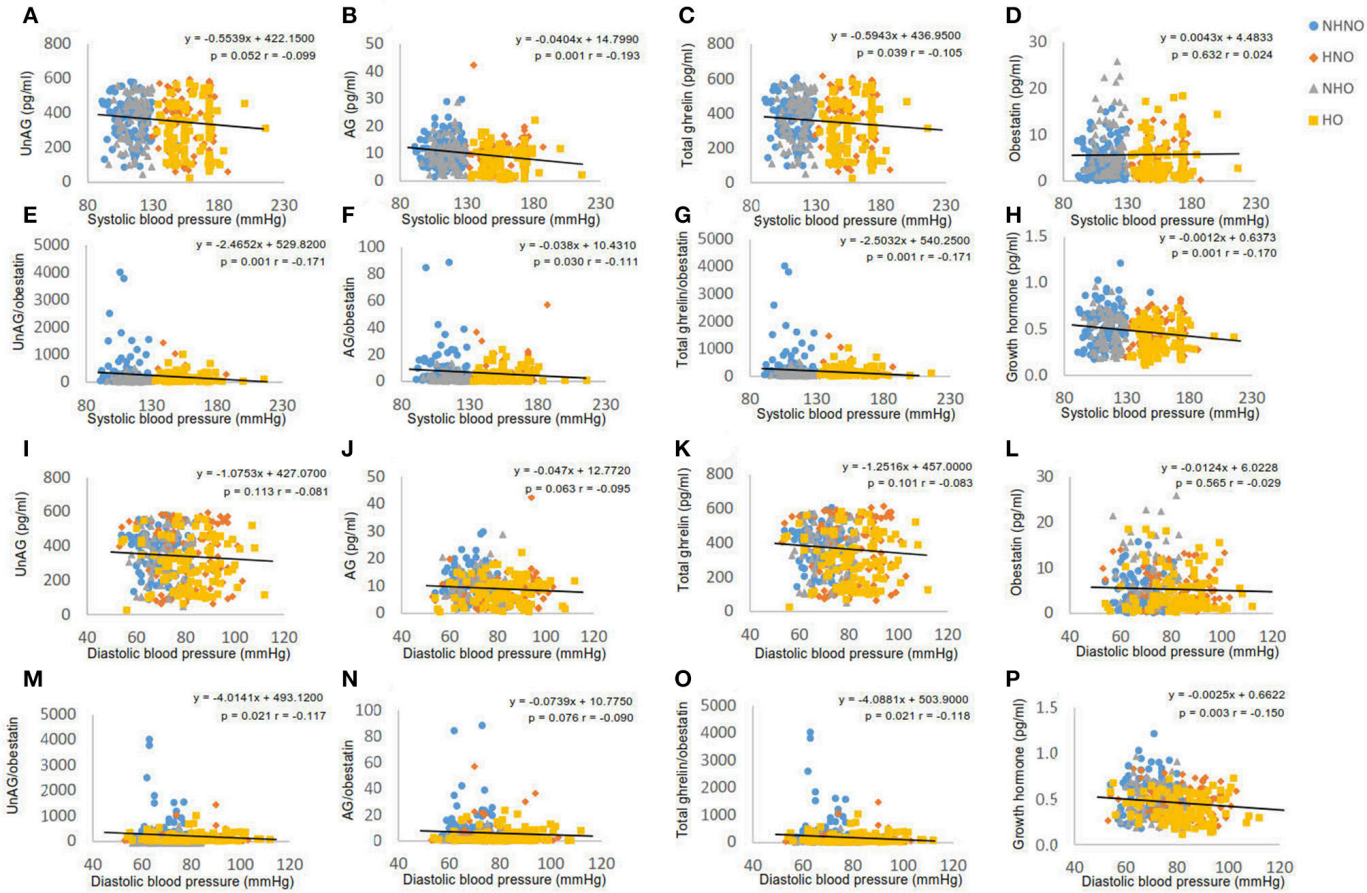
Correlation analyses of blood pressure to ghrelin gene products, growth hormone and ratios of two forms of ghrelin to obestatin. **(A)** Correlation between systolic blood pressure and serum level of unacylated ghrelin. **(B)** Correlation between systolic blood pressure and serum level of acylated ghrelin. **(C)** Correlation between systolic blood pressure and serum level of total ghrelin. **(D)** Correlation between systolic blood pressure and serum level of obestatin. **(E)** Correlation between systolic blood pressure and the ratio of unacylated ghrelin to obestatin in serum. **(F)** Correlation between systolic blood pressure and the ratio of acylated ghrelin to obestatin in serum. **(G)** Correlation between systolic blood pressure and the ratio of total ghrelin to obestatin in serum. **(H)** Correlation between systolic blood pressure and serum level of growth hormone. **(I)** Correlation between diastolic blood pressure and serum level of unacylated ghrelin. **(J)** Correlation between diastolic blood pressure and serum level of acylated ghrelin. **(K)** Correlation between diastolic blood pressure and serum level of total ghrelin. **(L)** Correlation between diastolic blood pressure and serum level of obestatin. **(M)** Correlation between diastolic blood pressure and the ratio of unacylated ghrelin to obestatin in serum. **(N)** Correlation between diastolic blood pressure and the ratio of acylated ghrelin to obestatin in serum. **(O)** Correlation between diastolic blood pressure and the ratio of total ghrelin to obestatin in serum. **(P)** Correlation between diastolic blood pressure and serum level of growth hormone. Pearson's correlation analyses were performed. Statistical significance was accepted at *p* < 0.05. Circle refers to the data of non-hypertensive non-centrally obese subjects. Rhombus refers to the data of hypertensive non-centrally obese subjects. Triangle refers to the data of non-hypertensive centrally obese subjects. Square refers to the data of hypertensive centrally obese subjects.

It was observed that systolic blood pressure was negatively correlated with AG (*p* = 0.001, *r* = −0.193) (Figure 2B), total ghrelin (*p* = 0.039, *r* = −0.105) (Figure 2C), GH (*p* = 0.001, *r* = −0.170) (Figure 2H), AG/obestatin ratio (*p* = 0.030, *r* = −0.111) (Figure 2F), UnAG/obestatin ratio (*p* = 0.001, *r* = −0.171) (Figure 2E), and total ghrelin/obestatin ratio (*p* = 0.001, *r* = −0.171) (Figure 2G). UnAG (Figure 2A) and obestatin (Figure 2D) did not exhibit any correlation with systolic blood pressure. Diastolic blood pressure exhibited correlations with UnAG/obestatin ratio (*p* = 0.021, *r* = −0.117) (Figure 2M), total ghrelin/obestatin ratio (*p* = 0.021, *r* = −0.118) (Figure 2O), and GH (*p* = 0.003, *r* = 0.150) (Figure 2P). UnAG (Figure 2I), AG (Figure 2J), total ghrelin (Figure 2K) and obestatin (Figure 2L) did not exhibit correlation with diastolic blood pressure.

**Figure 3:**
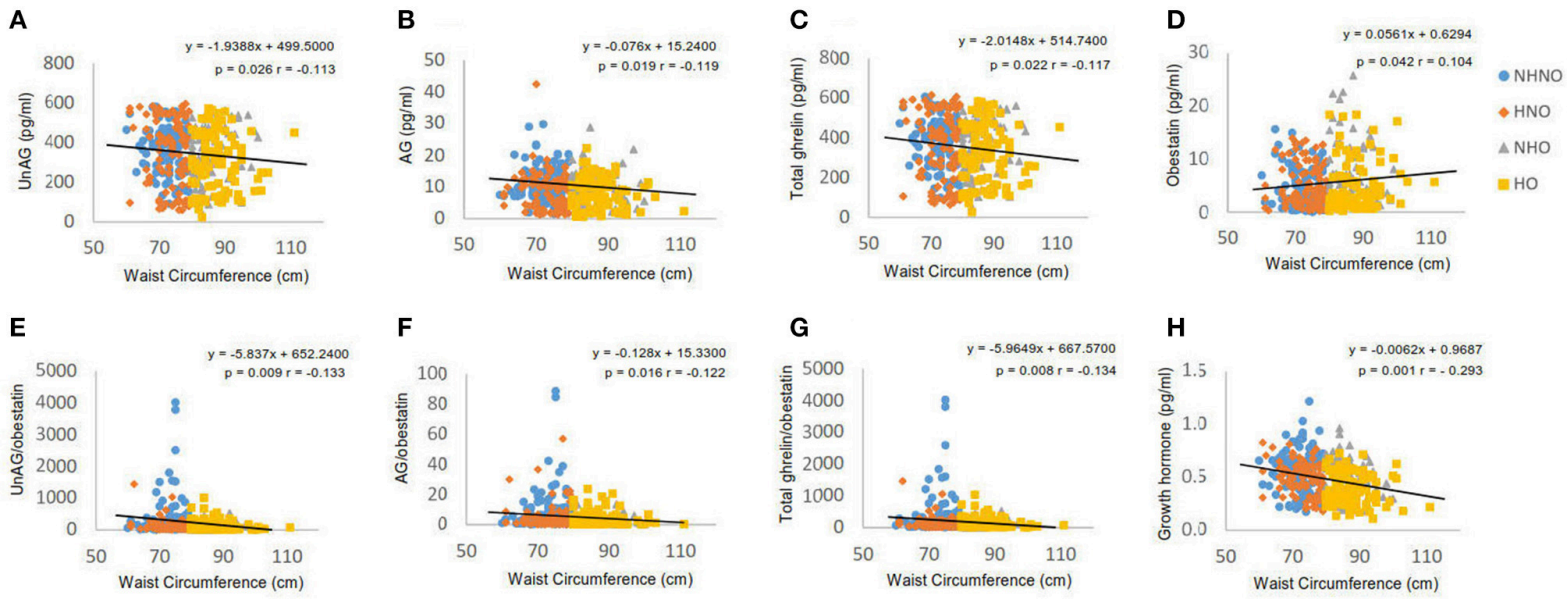
Correlation analyses of waist circumference to ghrelin gene products, growth hormone and ratios of two forms of ghrelin to obestatin. **(A)** Correlation between waist circumference and serum level of unacylated ghrelin. **(B)** Correlation between waist circumference and serum level of acylated ghrelin. **(C)** Correlation between waist circumference and serum level of total ghrelin. **(D)** Correlation between waist circumference and serum level of obestatin. **(E)** Correlation between waist circumference and the ratio of unacylated ghrelin to obestatin in serum. **(F)** Correlation between waist circumference and the ratio of acylated ghrelin to obestatin in serum. **(G)** Correlation between waist circumference and the ratio of total ghrelin to obestatin in serum. **(H)** Correlation between waist circumference and serum level of growth hormone. Pearson's correlation analyses were performed. Statistical significance was accepted at *p* < 0.05. Circle refers to the data of non-hypertensive non-centrally obese subjects. Rhombus refers to the data of hypertensive non-centrally obese subjects. Triangle refers to the data of non-hypertensive centrally obese subjects. Square refers to the data of hypertensive centrally obese subjects.

Waist circumference was negatively correlated with UnAG (*p* = 0.026, *r* = −0.113) (Figure 3A), AG (*p* = 0.019, *r* = −0.119) (Figure 3B), total ghrelin (*p* = 0.022, *r* = −0.117) (Figure 3C), GH (*p* = 0.001, *r* = −0.293) (Figure 3D), obestatin (*p* = 0.042, *r* = 0.104) (Figure 3E), AG/obestatin ratio (*p* = 0.016, *r* = −0.122) (Figure 3F), UnAG/obestatin ratio (*p* = 0.009, *r* = −0.133) (Figure 3G), and total ghrelin/obestatin ratio (*p* = 0.026, *r* = −0.134) (Figure 3H).

### Obestatin, but not ghrelin or GH, revealed the interaction of hypertension and central obesity

**Figure 4:**
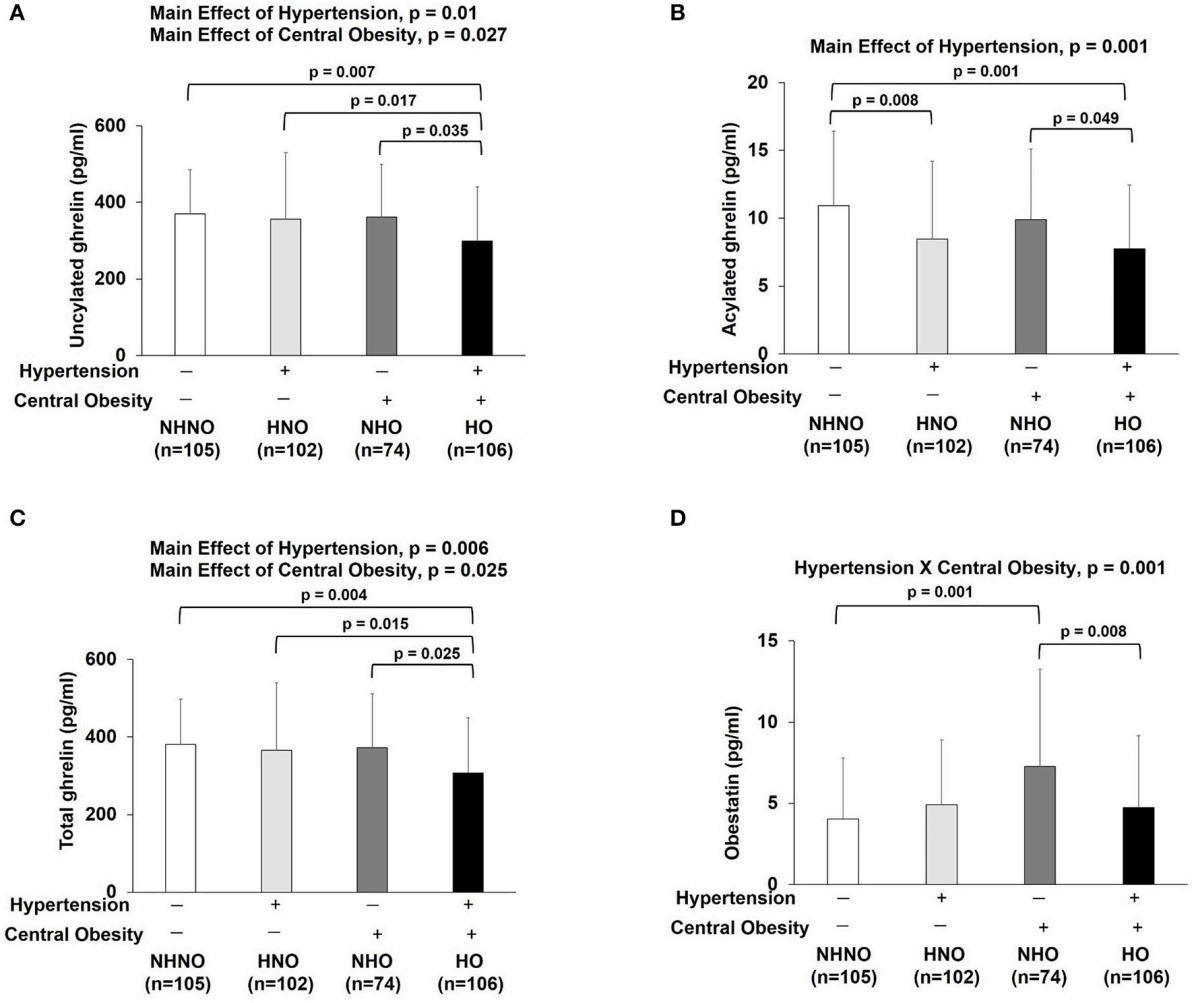
Ghrelin gene products: unacylated ghrelin, acylated ghrelin, total ghrelin and obestatin. There were significant main effects of hypertension and central obesity on unacylated ghrelin and total ghrelin **(A,C)**. There was significant main effect of hypertension on acylated ghrelin **(B)**. Unacylated ghrelin, acylated ghrelin and total ghrelin were decreased in subjects with hypertension and central obesity **(A–C)**. Acylated ghrelin also decreased in subjects with hypertension **(B)**. Obestatin revealed the interaction of hypertension with central obesity **(D)**. Obestatin increased in subjects with only central obesity but not in subjects with hypertension and central obesity **(D)**. The main effects of hypertension and central obesity and the interaction of hypertension with central obesity were analyzed with generalized estimating equations. Significance level was set at *p* < 0.05.

No significant interaction effect of hypertension and central obesity was found on UnAG, AG and total ghrelin (Figures 4A–C). Significant main effects of hypertension (*p* = 0.010) and central obesity on UnAG were identified (*p* = 0.027) (Figure 4A). Pairwise comparison showed that the UnAG in HO group was 20, 16, and 17% significantly lower than that of NHNO (mean differences = 70.18 pg/ml, *p* = 0.007), HNO (mean differences = 57.11 pg/ml, *p* = 0.017) and NHNO (mean differences = 62.65 pg/ml, *p* = 0.035), respectively. Significant main effect of hypertension was observed on AG (*p* = 0.001). Pairwise comparison suggested that lower circulating AG was associated with hypertension, regardless of the manifestation of central obesity. It was observed that the level of AG was 23% significantly lower in HNO group than that of NHNO (mean differences = 2.47 pg/ml, *p* = 0.008) and 22% significantly lower in HO group compared with the NHO group respectively (mean differences = 2.17 pg/ml, *p* = 0.049) (Figure 4B). The level of AG in HO was 29% significantly lower than that of NHNO (mean differences = 3.18 pg/ml, *p* = 0.001) (Figure 4B). There were significant main effects of hypertension (*p* = 0.006) and central obesity (*p* = 0.025) on the total level of ghrelin (Figure 4C). Pairwise comparison showed that total ghrelin in HO group was significantly lower compared with the other three groups. The total ghrelin in HO group was 19, 16, and 17% lower than that of NHNO (mean differences = 73.36 pg/ml, *p* = 0.004), HNO (mean differences = 57.82 pg/ml, *p* = 0.015) and NHO (mean differences = 64.83 pg/ml, *p* = 0.025), respectively.

Significant interaction effect of hypertension and central obesity was found on obestatin (*p* = 0.001) (Figure 4D), suggesting that obestatin is associated with blood pressure and waist circumference in an opposite manner. It was observed that the obestatin level in NHO was 79% significantly higher compared with that in NHNO (mean differences = 3.21 pg/ml, *p* = 0.001), while the obestatin level was 35% significantly lower in HO compared with that of NHO (mean differences = 2.52 pg/ml, *p* = 0.008) (Figure 4D).

### Ratios of ghrelin gene products, but not GH, reflected the interaction of hypertension and central obesity

**Figure 5:**
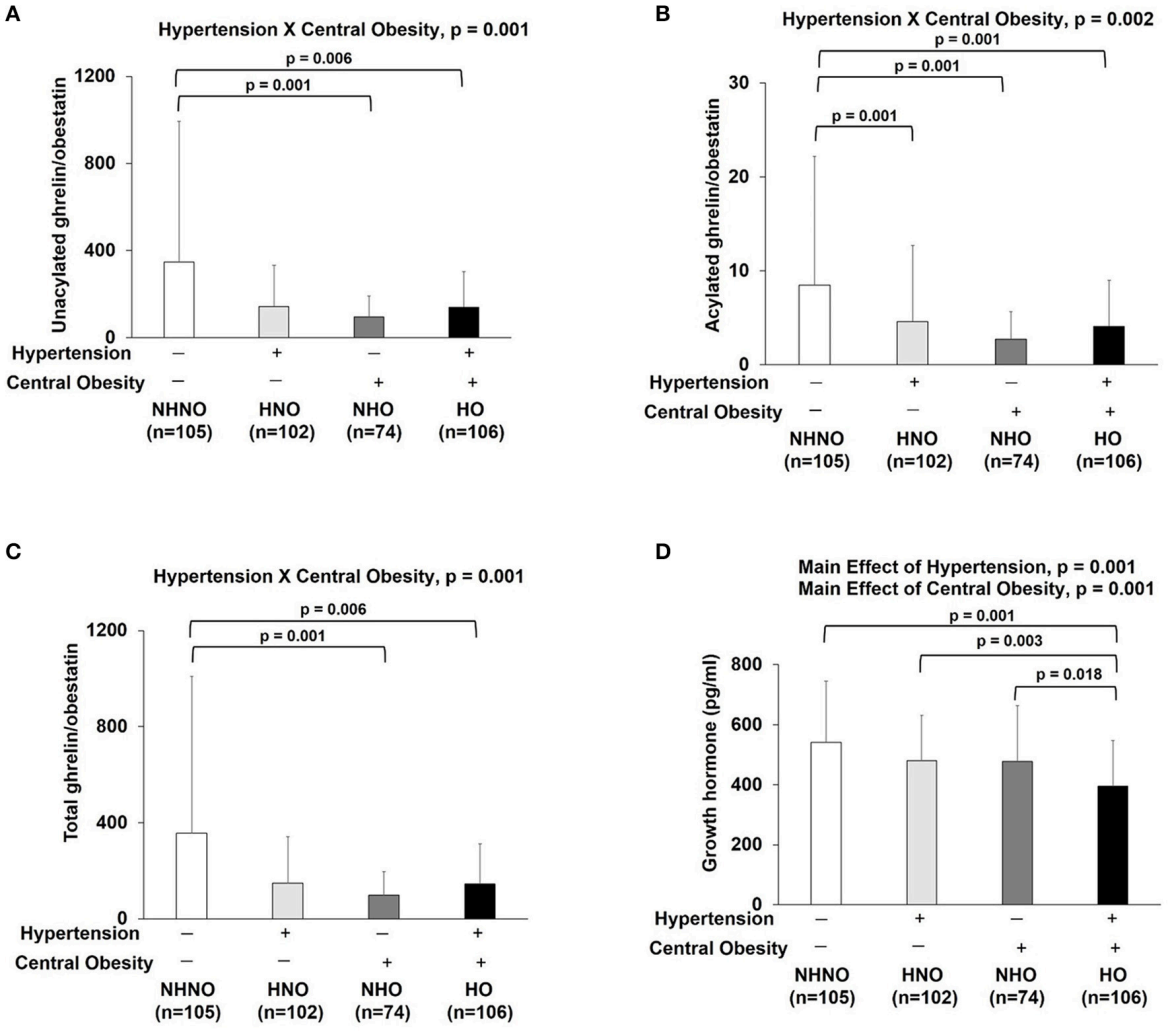
Ratios of ghrelin gene products (unacylated ghrelin/obestatin, acylated ghrelin/obestatin and total ghrelin/obestatin) and growth hormone. There was significant interaction of hypertension with central obesity on unacylated ghrelin/obestatin, acylated ghrelin/obestatin and total ghrelin/obestatin ratios **(A–C)**. The ratios of unacylated ghrelin/obestatin and total ghrelin/obestatin were decreased in subjects with only central obesity and subjects with both hypertension and central obesity **(A,C)**. The ratio of acylated ghrelin/obestatin was decreased in subjects with hypertension, central obesity and both hypertension and central obesity **(B)**. There were significant main effects of hypertension and central obesity on growth hormone **(D)**. Growth hormone was decreased in subjects with hypertension and central obesity **(D)**. The main effects of hypertension and central obesity and the interaction of hypertension with central obesity were analyzed with generalized estimating equations. Significance level was set at *p* < 0.05.

Significant interaction effects of hypertension and central obesity were found on UnAG/obestatin (*p* = 0.001) (Figure 5A), AG/obestatin (*p* = 0.002) (Figure 5B), and total ghrelin/obestatin ratios (*p* = 0.001) (Figure 5C). The UnAG/obestatin ratio in NHNO group was 265 and 147% significantly higher than that of NHO (mean differences = 252.0, *p* = 0.001) and HO (mean differences = 206.7, *p* = 0.001) (Figure 5A), respectively. Similar patterns were observed in the total ghrelin/obestatin ratio (Figure 5C). The NHNO group manifested higher total ghrelin/obestatin ratio than the NHO (mean differences = 211.1, *p* = 0.001) and HO groups (mean differences = 257.7, *p* = 0.006) (Figure 5C), respectively. The AG/obestatin ratio in NHNO group was 263, 207, and 107% significantly higher than that of HNO (mean differences = 3.9, *p* = 0.012), NHO (mean differences = 5.7, *p* = 0.001) and HO groups (mean differences = 4.4, *p* = 0.002) (Figure 5B).

No significant interaction effect of hypertension and central obesity was found on GH (Figure 5D). However, there were significant main effects of hypertension (*p* = 0.001) and central obesity (*p* = 0.001) on GH (Figure 5D). *Post hoc* analyses showed that the GH level in HO group was 27, 18, and 17% significantly lower when compared to NHNO group (mean difference = 0.146 pg/ml, *p* = 0.001), HNO group (mean difference = 0.085 pg/ml, *p* = 0.003), and NHO group (mean difference = 0.082 pg/ml, *p* = 0.018), respectively (Figure 5D).

## Discussion

Hypertension and obesity are two of the leading cardiovascular epidemics that lead to devastating health consequences. A cross-sectional survey involving data from the Third National Health and Nutrition Examination Survey in the United States of America estimated that more than 45% obese individuals (Body Mass Index ≥ 30) were hypertensive. It has also been proposed that severe obesity (Body Mass Index ≥ 40) contributed to approximately 60–70% of hypertension in adults, (33). To develop novel strategies for the management of hypertension and obesity, it is necessary to identify the mechanisms underlying obesity-related hypertension. The relationships between ghrelin and obesity alone (16, 17) and that with hypertension alone (18) are well-documented. However, studies addressing the regulation of ghrelin gene products under the interaction between hypertension and obesity are scarce. Although previous studies have unanimously reported that altered circulating levels of ghrelin and obestatin are associated with hypertension and obesity, it is noteworthy that the form of ghrelin was not specified in most of the previous investigations. In this study, we aimed to address the changes of both ghrelin forms, obestatin, GH, and the ratios of the two forms of ghrelin to obestatin in the presence of the interaction between hypertension and central obesity. We have demonstrated for the first time that the circulatory levels of several ghrelin gene products and the ratios of total ghrelin/obestatin, AG/obestatin, and UnAG/obestatin are correlated with concomitant manifestation of hypertension and central obesity.

In addition to the observation that hypertensive individuals were older, our correlation analyses indicated that age was positively correlated to systolic blood pressure and negatively correlated with AG. It is also observed that AG was negatively correlated with systolic blood pressure, suggesting that the decrease in AG in aged individuals may be a plausible mechanism of age-related hypertension. We have demonstrated that growth hormone and the ratios of UnAG/obestatin and total ghrelin /obestatin are associated with systolic blood pressure, diastolic blood pressure, and waist circumference. We have also observed correlation of particular ghrelin gene products to the cardiovascular risk factors. These data suggested that there are linkages between these hormones and the cardiovascular risk factors. Both main effects of hypertension and central obesity were observed for UnAG, total ghrelin and GH. *Post hoc* analyses showed that decreases in UnAG, total ghrelin and GH were only observed in centrally obese, hypertensive women, but not in the women with central obesity or hypertension alone. These data suggested that high blood pressure and central obesity might synergistically reduce further the levels of UnAG, total ghrelin and GH. Our findings are in line with previous studies exhibiting that total plasma ghrelin was lower in individuals with both hypertension and obesity in comparison to those obese or healthy control (19). According to our data, the decrease in total ghrelin of centrally obese, hypertensive women was largely accounted by the reduction of UnAG, which is the predominant form of circulatory ghrelin (34). Only main effect of hypertension was observed on AG, suggesting that hypertension is sufficient to reduce the level of AG, regardless of the presentation of central obesity. Administration with AG has been shown to decrease the blood pressure (22), implying that a decrease in AG may possibly involve in the development of hypertension. Notably, our findings have indicated that the levels of UnAG, AG and total ghrelin were the lowest in women with both hypertension and central obesity among all the studied groups, suggesting that a low ghrelin profile may represent a biochemistry abnormality in obesity-related hypertension.

In contrast, efforts studying the association between obestatin and obesity/hypertension have yielded mixed results (13). We observed that hypertension alone elevated the level of obestatin by 22%; although this did not reach to statistical significance, while centrally obese, normotensive women manifest a significantly higher level of obestatin compared with their non-obese, normotensive counterparts. This data suggested that obestatin level is increased by the presence of hypertension or central obesity alone. Interestingly, the obestatin level in women with both central obesity and hypertension was not the highest among the four groups; rather was significantly lower than that of centrally obese, normotensive counterparts. Our findings are indeed in accordance with a previous study showing that obestatin was significantly lower in centrally obese, hypertensive individuals compared with those with central obesity alone (19).The observed interaction between hypertension and central obesity in this study suggested that hypertension and central obesity may confer opposing effects on obestatin. This observation warrants future investigation to reveal whether obestatin represents a compensatory mechanism to partially maintain metabolism or homeostasis in a similar manner as in individuals with neither hypertension nor central obesity.

Our results indicated that alteration of all three ghrelin gene products may present implications in hypertension and central obesity. As the ghrelin gene products are functionally distinct (8, 10), examining the changes in the ratios of various forms of ghrelin/obestatin can provide further insights on how hypertension and central obesity affect the balance of various ghrelin forms. While a decreased total ghrelin/obestatin ratio in women with obesity has been previously reported (16), to our best understanding, we have provided the first piece of evidence that the presentation of both hypertension and central obesity is associated with decreased UnAG/obestatin, AG/obestatin and total ghrelin/obestatin ratios. Total ghrelin/obestatin ratio has been demonstrated to correlate negatively with body mass index, waist circumference, waist/hip ratio and insulin (16). More recently, AG/obestatin ratio was shown to be significantly lower in children and adolescents with obesity compared to their normal-weight counterparts (35). In addition, the total ghrelin/obestatin ratio was also reduced in hypertensive individuals compared with normotensive control (18). Not only are our findings in congruent with previous studies, we have also reported, for the first time, that hypertension does not decrease further the ratios of UnAG/obestatin, AG/obestatin and total ghrelin/obestatin ratios in conjunction with obesity. It is worth-noting that these ratios are the lowest in centrally obese, normotensive individuals; but not in those harboring both conditions. This observation suggested that the reductions of various ghrelin form/obestatin might not necessarily correlate with an improved metabolic status.

Given that AG stimulates the release of GH from the anterior pituitary (36), GH may mediate the effects of AG in the regulation of blood pressure and body fat as demonstrated in our correlation analyses. GH intervention has been shown to attenuate adverse prenatal environment-induced obesity and hypertension in rats (21, 37). Earlier studies have also reported that spontaneous and induced GH secretion was decreased in obesity (38), whereas GH supplementation in GH-deficient adults resulted in a reduction of central obesity (39). This is the first attempt to demonstrate that GH, UnAG and total ghrelin are concordantly decreased in individuals with hypertension and central obesity compared to their normotensive, non-centrally obese counterparts. AG is the only form of ghrelin that has been proven to physiologically stimulate the release of GH from the anterior pituitary. Interestingly, GH was regulated in a similar manner as with UnAG, but not AG under the interaction of hypertension and central obesity. While AG was decreased with hypertension regardless of central obesity, GH was decreased only with hypertension and central obesity. Moreover, we speculated that the reduced GH in the centrally obese-only group could be an effect secondary to the reduced half-life, frequency and daily production rate of GH (38). Although the signaling molecules downstream of UnAG need to be identified, our results suggested that UnAG might indirectly regulate GH via unknown signaling pathways. Furthermore, the responses of GH-regulating hormones to hypertension and/or obesity should be addressed in the future studies.

Unlike most studies which only investigated the relationship between total ghrelin and obesity/hypertension alone, we have performed detailed analyses of various ghrelin gene peptides and GH in individuals manifesting both central obesity and hypertension. Our study demonstrated that the presence of hypertension alone is associated with decreased level of AG, while the presence both hypertension and central obesity is associated with the decreased circulatory UnAG. Nonetheless, interaction effect between hypertension and central obesity was observed with obestatin and the ratios of the two ghrelin forms to obestatin, suggesting that the obestatin and the ratios of the two ghrelin forms to obestatin were not augmented or even dampened in response to the co-manifestation of hypertension and central obesity compared with the presence of hypertension or central obesity alone. Most importantly, our reported changes in ghrelin gene products and GH indicated that a complete profile of ghrelin gene products, rather than the level of total ghrelin or any of its product alone, is necessary to better assess the metabolic health of an individual. It is thus tempting to study the interaction among a more diverse combination of cardiovascular risk factors to understand how the ghrelin gene products are regulated under complicated metabolic disorders. Our data suggested that a more comprehensive understanding on the interacting influence of different cardiovascular risk factors on ghrelin gene products is needed for the future development of therapeutic agents on managing energy homeostasis and cardiovascular functions.

## Conclusion

Not only the associations between hypertension or obesity alone with circulating ghrelin gene products and GH were revealed, this study demonstrated that various ghrelin gene products and GH are regulated in different manners under the interaction between hypertension and central obesity compared with that the presence of hypertension or central obesity alone.

## Author contributions

AY contributed to data analysis, analysis of the paper, and writing of the paper. FU contributed to study design, data collection, data analysis, analysis of the paper, and writing of the paper. BT contributed to data collection. PL contributed to data analysis and analysis of the paper. CL and CW contributed to analysis of the paper. PS contributed to study design, analysis of the paper, and writing of the paper. All authors approved the final article.

### Conflict of interest statement

The authors declare that the research was conducted in the absence of any commercial or financial relationships that could be construed as a potential conflict of interest.
